# Carotid Cavernous Fistula and Its Association With Spine Surgery: A Case Report

**DOI:** 10.7759/cureus.75160

**Published:** 2024-12-05

**Authors:** Brandon Sharkey, Ethan Kosco, Andrew Waack, Vito Lucarelli, Brandon C Gabel

**Affiliations:** 1 Division of Neurosurgery, The University of Toledo College of Medicine and Life Sciences, Toledo, USA; 2 Division of Neurosurgery, Department of Surgery, The University of Toledo College of Medicine and Life Sciences, Toledo, USA; 3 Department of Neurosurgery, ProMedica Toledo Hospital, Toledo, USA

**Keywords:** angiography, carotid cavernous fistula, cerebrospinal fluid leak, cranial nerve palsy, dural tear, magnetic resonance imaging

## Abstract

A carotid cavernous fistula (CCF) is a disruption in the carotid arteries within the cavernous sinus. The pooling of blood in the sinus causes a myriad of neurological deficits. When correctly diagnosed, this condition can be easily managed through surgical intervention. However, its rareness and similarity in presentation to other neurological conditions allow for misdiagnosis and subsequent progression to permanent disability. Therefore, it is imperative to understand the unique clinical features and signs of CCFs. We report on the clinical workup, diagnosis, and treatment of the first documented case of a patient who developed a carotid cavernous fistula in conjunction with spine surgery.

## Introduction

Carotid cavernous fistulas are a serious condition that involves spontaneous or trauma-induced disruption in the carotid arteries within the cavernous sinus. This disruption causes blood to leak into the sinus, which impedes the function of cranial nerves III, IV, V1, V2, and VI [1]. Early signs of CCF often include proptosis, glaucoma, severe headaches, partial blindness, and conjunctival hyperemia, especially in patients with preexisting conditions such as atherosclerosis, hypertension, and diabetes. If left untreated, patients can develop periorbital disfigurement and permanent vision loss [2]. Despite effective methods of diagnosis using angiography and blood vessel repair, this condition is often misdiagnosed due to it making up only 1.5% of endovascular-treated ruptured aneurysms and its similarity in presentation to cerebrospinal fluid (CSF) leaks [1-3]. Therefore, CCFs have been under-investigated, and additional research is required to delineate the cause, unique features, and proper treatment of this phenomenon. Often misdiagnosed due to its ambiguous presentation, we present a successful clinical workup and treatment of a 78-year-old female patient who developed a CCF in the setting of spinal fusion. We suggest a potential relationship between prolonged prone positioning during surgical intervention and the occurrence of CCF.

## Case presentation

**Figure 1:**
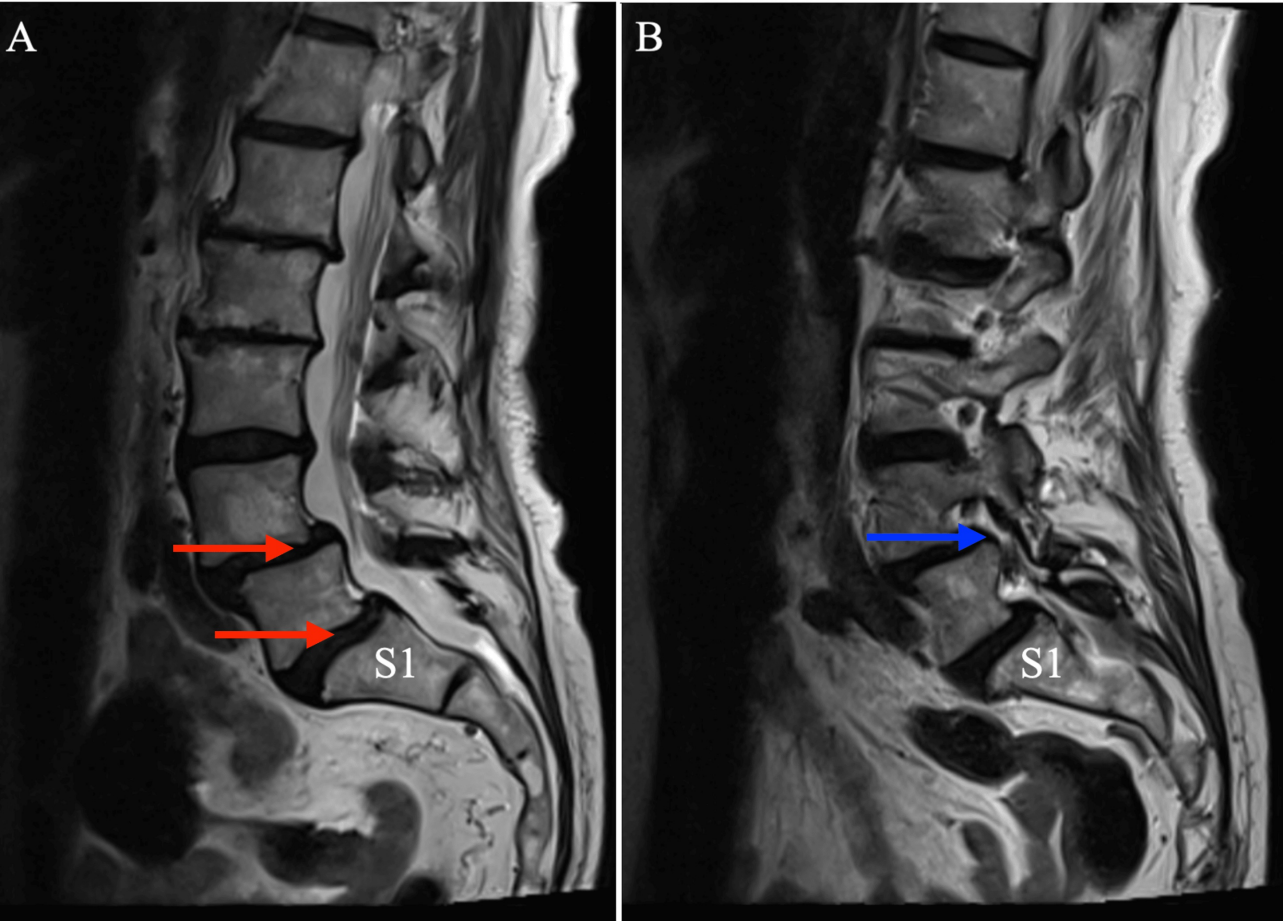
Pre-operative lumbar T2 sagittal MRI demonstrating significant degenerative spondylosis of L4-S1 levels (A), and severe lateral stenosis at L4-L5 (B). The S1 vertebrae is labeled "S1." The L4-S1 degenerative change is highlighted with red arrows and the lateral stenosis is delineated with a blue arrow.

A 78-year-old female patient presented for bilateral lateral radicular leg discomfort and back pain refractory to conservative management including physical therapy and anti-inflammatory medications. Magnetic resonance imaging (MRI) of the lumbar spine revealed significant degenerative spondylosis of L4-S1 levels including severe lateral recess stenosis at L4-L5 and bilateral pars defects at L5-S1 (Figure 1). She was neurologically intact with no remarkable findings on physical exam. Operative management was pursued due to her symptoms and imaging findings. She underwent a successful L4-S1 posterior instrumented fusion with partial laminectomies at L4 and L5 along with an L4-L5 bilateral facetectomy. There was no significant intraoperative fluid leak. Estimated blood loss was minimal, and the procedure lasted approximately 228 minutes with the patient prone on a Jackson spine table.

The patient’s postoperative course was uneventful until postoperative day 3 when the patient developed severe throbbing headaches and neck and shoulder pain/stiffness that resolved with laying flat. The patient’s symptoms resolved the following day, so she was discharged. Two weeks following her surgery, she presented for suture removal and had no complaints of headaches or neck and shoulder pain.

**Figure 2:**
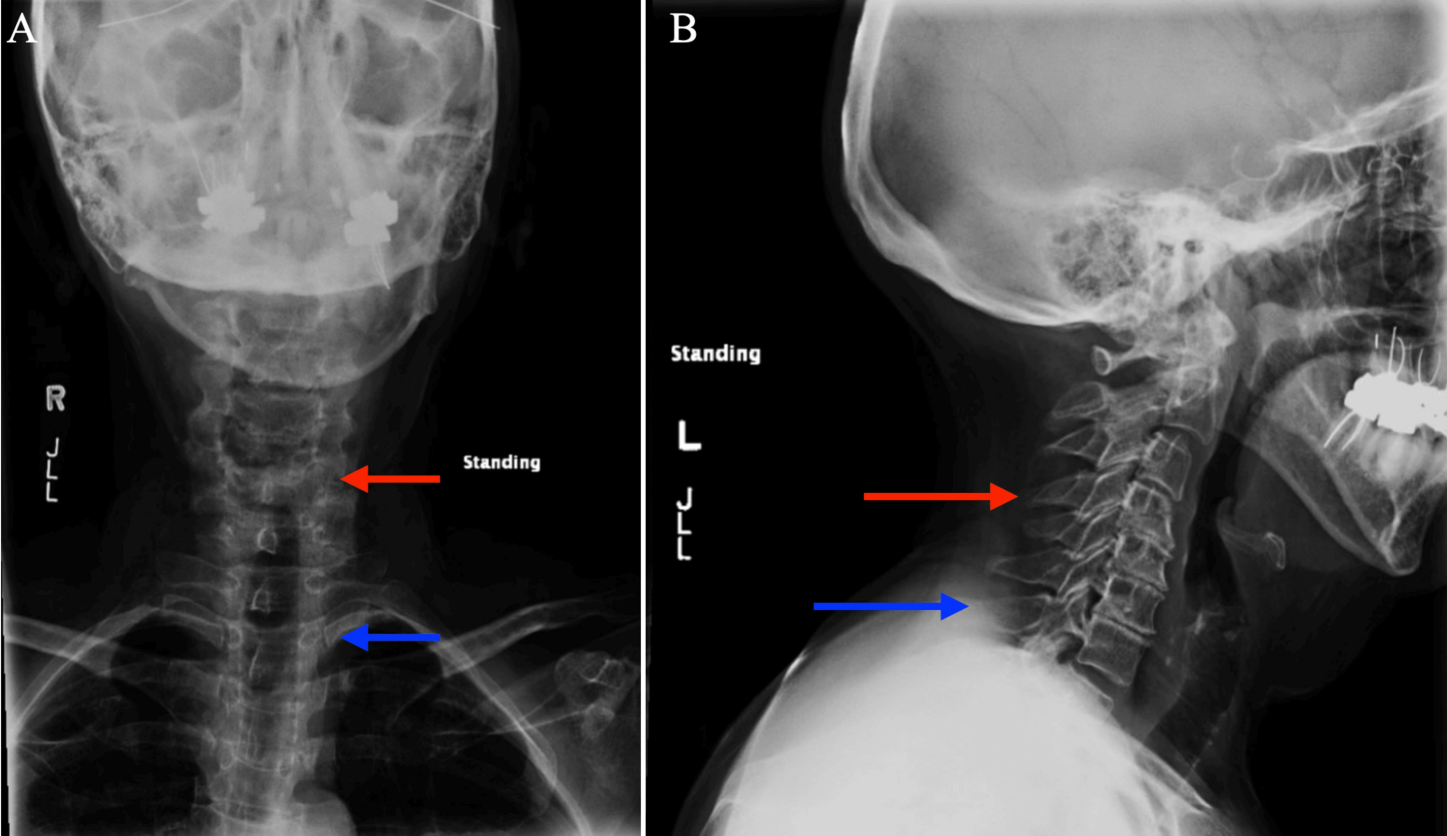
Coronal (A) and sagittal (B) x-ray images of the cervical spine demonstrating degenerative changes from C4 through C7. The red arrows mark the C4 vertebrae and the blue arrows mark the C7 vertebrae.

Six weeks following the surgery, the patient presented for a follow-up with complaints of bifrontal headaches that were worse in the morning for the past two weeks. She also reported neck discomfort and sharp discomfort whenever she turned her head to one side or extended her neck. The headaches were not positional nor particularly severe. There was no evidence of a spinal fluid leak from the incision nor palpable fluid collection appreciated on the physical exam. An x-ray of the cervical spine demonstrated degenerative changes from C4 through C7 with complete loss in disc space height at each level from C4 inferiorly (Figure 2).

**Figure 3:**
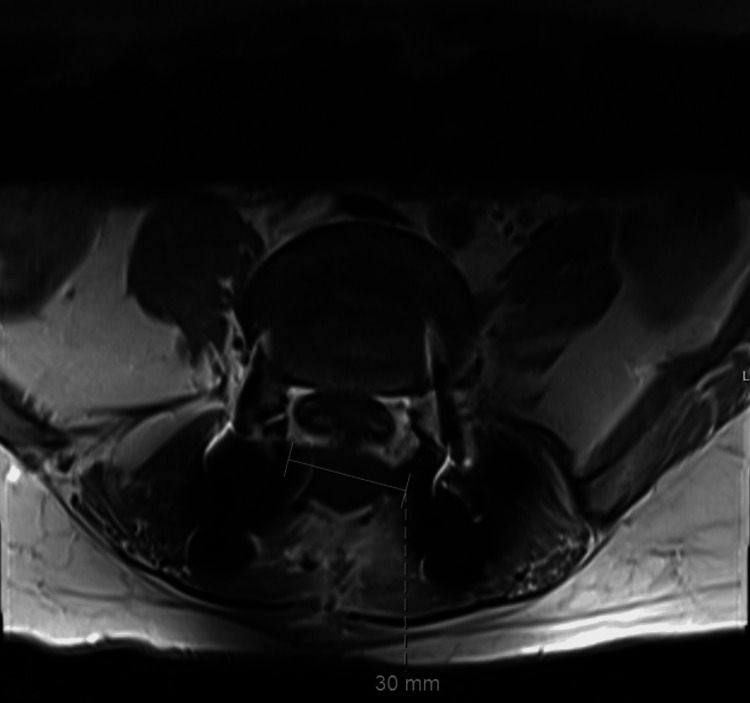
Follow-up MRI lumbar T1 axial demonstrating a 30 mm peripherally enhancing fluid collection along the surgical defect at the native abdominal location.  Fluid accumulation is labeled with the measuring tool.

The patient presented to the Emergency Department on two separate occasions in the following two weeks; first, she had a pounding headache in bilateral temples with swelling below her left eye and an episode of emesis the evening prior. She was also noted to have conjunctivitis in both eyes and was given antibiotic eye drops. She presented the second time with complaints of a constant headache in the middle of the night, nausea, and new onset blurry vision in her left eye. The patient was also hypertensive with 170 systolic blood pressure. A brain CT scan showed no evidence of an acute intracranial process. Basic lab workup was unremarkable on both visits. The patient was discharged the second time with prescriptions for Percocet and Zofran and was recommended to follow up with her neurologist. Eight weeks following her fusion, the patient was struggling with worsening headaches and nausea. A lumbar spine MRI showed a fluid collection adjacent to the dura with a possible dural tear (Figure 3). Her worsening symptoms prompted a return to the operating room for wound exploration and repair of a suspected CSF leak. There was no apparent CSF leak. Valsalva was performed with no obvious egress of spinal fluid. Given that there may have been an occult non-identifiable leak the dura was covered with two layers of Surgicel (Ethicon, Johnson & Johnson, USA) followed by Adherus (HyperBranch Medical Technology, Durham, NC, USA) tissue sealant. A lumbar drain was placed through the next most rostral intact interspinous space brought out through a separate stab incision. A 10-round Jackson-Pratt drain was tunneled into the sub-fascial space and brought out through an additional stab incision.

**Figure 4:**
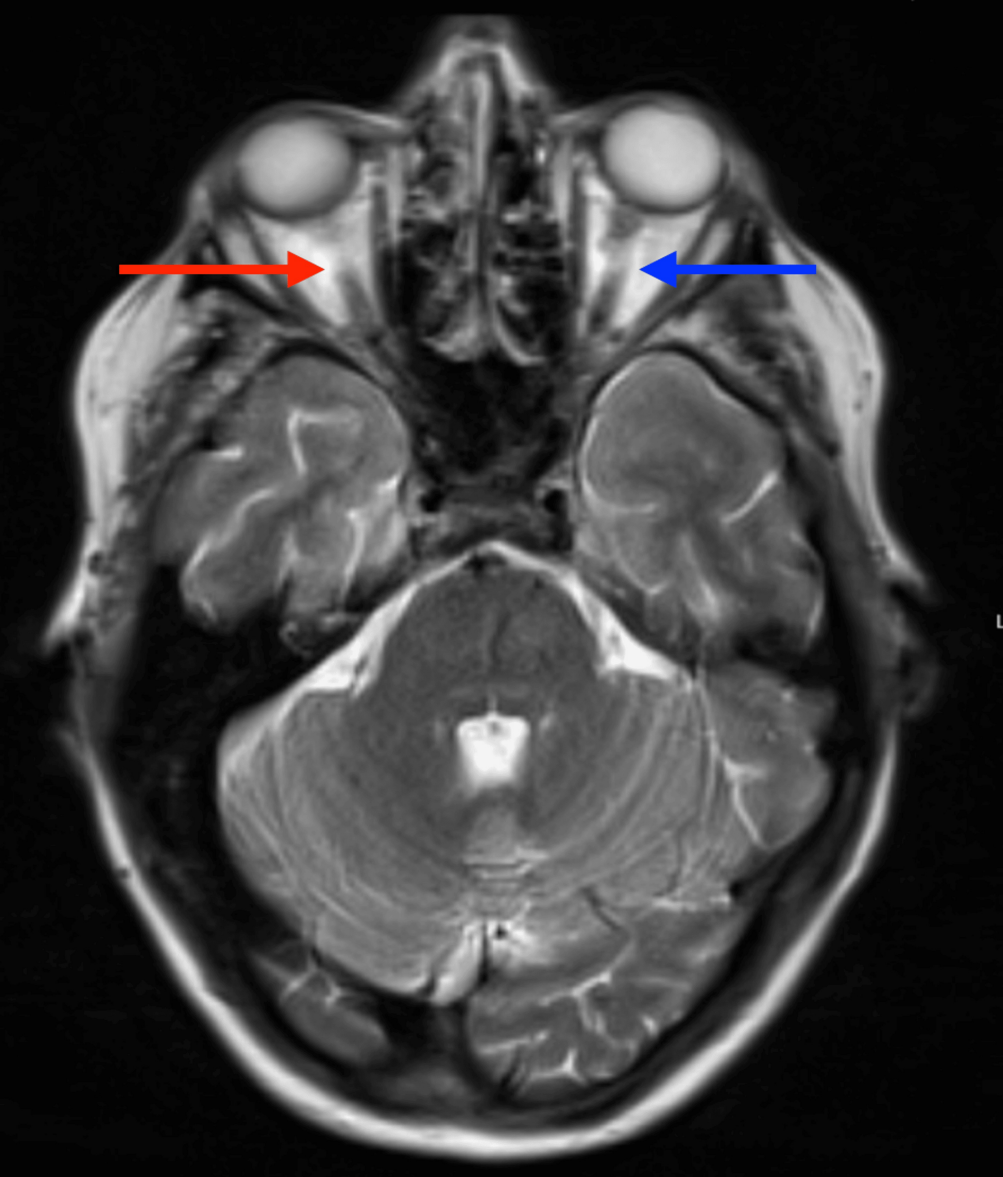
Brain MRI demonstrating prominent superior ophthalmic veins. The right ophthalmic vein (red arrow) is greater in size than the left (blue arrow).

Following the surgery, she had two episodes of emesis with continued headaches and nausea. However, she experienced significant improvement in her headaches and nausea over the subsequent days. She was discharged one week later following drain removal and complete resolution of her symptoms. Two weeks following discharge, she presented to the emergency department again for continued headaches, left conjunctival hemorrhage, mild proptosis, diplopia, and a cranial nerve (CN) III palsy. Given the unclear etiology for her headaches and new exam findings, there was a concern for Tolosa-Hunt syndrome or a carotid-cavernous fistula which prompted a brain MRI. Imaging demonstrated prominent superior ophthalmic veins, with the right being greater than the left (Figure 4).

**Figure 5:**
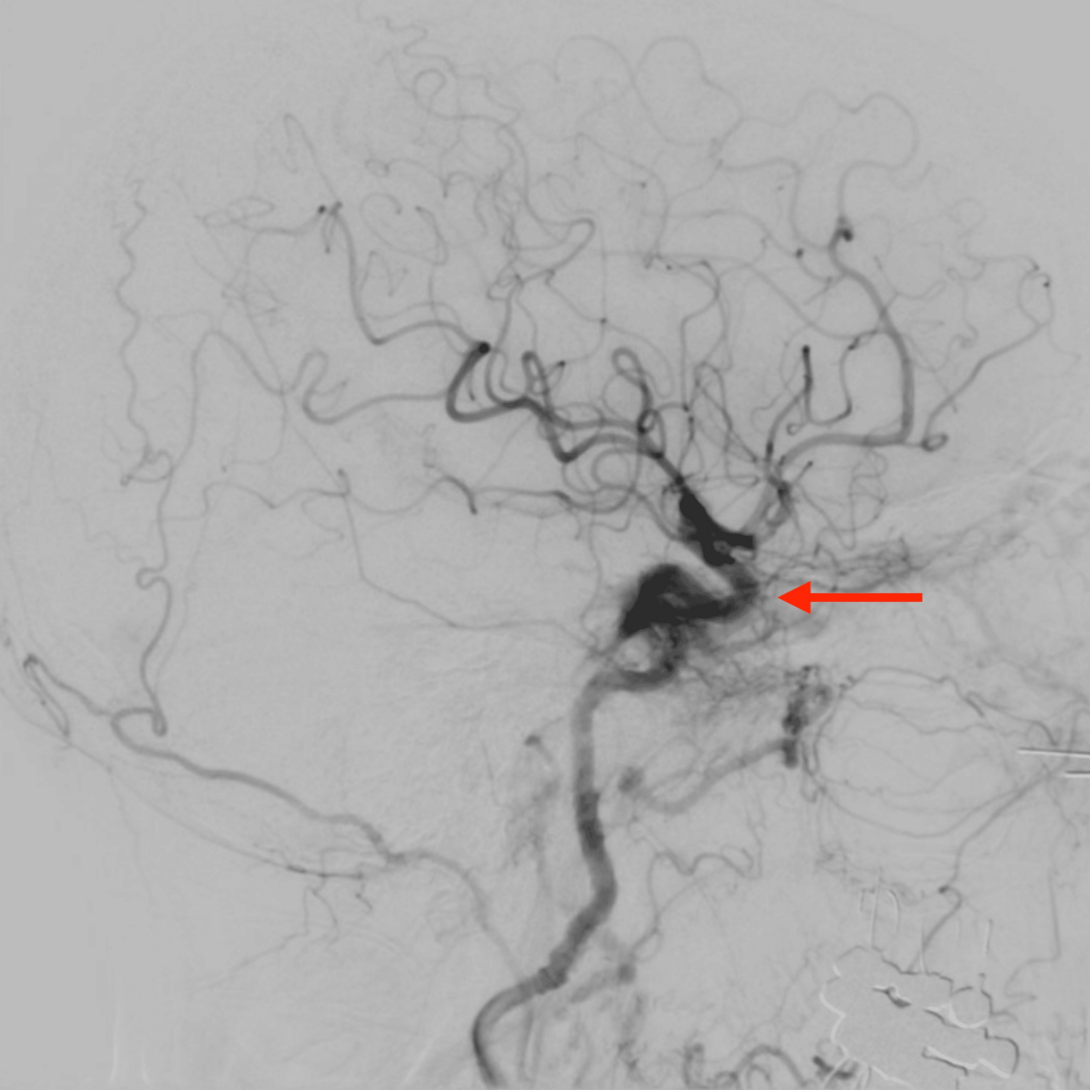
Diagnostic cerebral angiogram demonstrating fistula in the wall of the cavernous sinus on the left side (red arrow).

A left carotid-cavernous fistula was confirmed using diagnostic angiography. The patient underwent transvenous embolization for carotid-cavernous fistula in the intercavernous and left cavernous sinus. The fistula was nearly completely occluded with a significant reduction of flow into the fistula and gradual occlusion noted during angiography from the arterial supply (Figure 5). She had a successful coil embolization of a Barrow Type C carotid-cavernous fistula. Following the procedure, the patient had a significant improvement in her symptoms and was discharged three days later. Her postoperative course was significant for an improvement in her headaches and a lingering CN III and VI palsy predominantly of the left eye.

## Discussion

Carotid-cavernous fistulas (CCF) are pathological shunts between the cavernous sinus and the carotid artery or its meningeal branches. The Barrow classification system anatomically describes four types of CCFs. Type A CCFs are direct, high-flow communications between the cavernous sinus and the internal carotid artery (ICA) as it traverses the sinus. Low-flow fistulas indirectly connect the carotid artery to the cavernous sinus via the ICA meningeal branch (Type B), external carotid artery meningeal branch (Type C), or both branches (Type D) [1,2].

Several etiologies regarding the pathogenesis of CCF have been described. Type A CCFs are often associated with trauma, ruptured ICA aneurysms, connective tissue diseases, or iatrogenic injury [3,4]. Type A CCF has been reported after various procedures, particularly those involving direct carotid manipulation or involvement of adjacent structures; procedures reported in association with Type A CCF include carotid endarterectomy, mechanical thrombectomy, transsphenoidal exploration, craniotomy and ocular surgery [5-8]. Traumatic CCFs may occur as a result of bony fragment damage to the vessel wall secondary to basilar skull fracture or shear forces directly damaging the vessel wall [3]. Indirect CCFs most commonly affect elderly women and are classically associated with ICA dissection, hypertension, and connective tissue diseases, including atherosclerosis, fibromuscular dysplasia, and Ehlers-Danlos syndrome [4,8-12].

The presenting signs and symptoms of CCF are variable, depending on the characteristics of the CCF. Direct CCF often presents with conjunctival injection, blurred vision, headache, pulsating proptosis, and strabismus, most often affecting the sixth cranial nerve (the only cranial nerve traversing the interior of the cavernous sinus) [2,4]. Low-flow CCFs often follow an insidious course, and conjunctival injection is typically the predominant physical complaint [3]. Notably, low-flow CCFs are especially subject to misdiagnosis; our case represents a delayed diagnosis due to the patient’s recent spinal surgery and symptomatology mimicking a dural tear. The diagnosis of CCF is dependent upon imaging. Computed tomography (CT) and magnetic resonance (MR) imaging with or without angiography are often the first modalities utilized in assessing possible CCF and may be useful in diagnosing CCF; however, digital subtraction angiography (DSA) is currently considered the “gold standard” imaging modality for CCF because it demonstrates the cavernous sinus filling and drainage patterns [3]. Strong clinical suspicion or CT/MRI evidence of possible CCF should prompt DSA [3].

The exact treatment strategy is dependent upon the characteristics of the CCF. Direct CCFs are most often treated through endovascular approaches, with embolic materials, such as coils, acrylic glue, or ethylene vinyl alcohol copolymer, injected into the cavernous sinus to close fistula patency [3,4]. Endoluminal stenting may be utilized to prevent subsequent embolism and promote endothelial proliferation [3]. Twenty to sixty percent of indirect CCFs spontaneously resolve; thus, conservative management is usually attempted before intervention [2,3]. External manual carotid compression with the contralateral hand several times per day has been shown to promote fistula closure [4]. If surgical intervention is indicated, the same endovascular techniques used to repair direct CCFs are the first-line treatment modalities [4]. However, because indirect CCFs involve smaller, tortuous carotid branches, endovascular therapy is not as effective compared to direct CCFs [4]. Overall, endovascular treatment has a greater than 80% cure rate for CCF [3]. If first-line treatments fail, open surgical ICA ligation can be attempted [3]. Our patient was successfully treated with CCF coil embolization.

CCFs are easily misdiagnosed, as CCF presentations can be highly variable and may be similar to several different diseases [5]. Specific clinical histories may further confound the diagnosis of CCF. For example, Jain et al. describe a CCF that was initially misdiagnosed as hemorrhagic choroidal detachment following ocular surgery [5]. Similarly, our patient's history of recent spinal surgery made a CSF leak a more plausible diagnosis than CCF. CSF leaks can present very similarly to CCF, with headaches, nausea, neck or back tightness, and cranial nerve palsies, being commonly shared symptoms [6]. The diagnosis of CCF in our case was delayed because the patient’s recent surgical history made a dural tear more plausible. However, surgical exploration and drainage did not improve the patient’s symptoms, suggesting a dural tear was not present. After ruling out a dural tear, subsequent workup led to the diagnosis and treatment of CCF.

To our knowledge, there is no association between CCF and spinal surgery or prolonged periods of prone positioning. There have been no reported cases of CCF following surgery not involving the carotid artery or related structures; all reported iatrogenic causes of CCF involve direct carotid involvement or surgery to adjacent structures [9,7,10-12]. The patient has no known vascular diseases and suffered no recent trauma. Indirect CCFs (such as the Type C CCF seen in this patient) disproportionately affect elderly women [4,8]. We hypothesize that perhaps a prolonged period spent in the prone position during her spinal surgery is responsible for the CCF in a patient whose demographics predisposed her to CCF.

## Conclusions

Our case represents the first reported CCF occurring in conjunction with spine surgery. Although unsubstantiated, we believe there may be an association between a prolonged period in the prone position and the development of CCF. Furthermore, we would like to document our clinical workup; in which the clinical history and symptomatology suggested a dural tear, although subsequent workup led to the diagnosis of CCF. This case demonstrates a difficult diagnosis of CCF and its potential relationship to spinal surgery.
